# In-vivo Assessment of the Proximal Branches of the Anterior Cerebral Artery Using Rotational Angiography

**DOI:** 10.1007/s00062-025-01539-y

**Published:** 2025-07-22

**Authors:** Maximilian Rauch, Joachim Berkefeld, Janine Mokbel, Thomas Deller, Elke Hattingen, Stefan Weidauer

**Affiliations:** 1 Institute for Neuroradiology, Johann Wolfgang Goethe-University, Theodor Stern Kai 7, 60590 Frankfurt am Main, Germany; 2 Institute of Clinical Neuroanatomy, Dr. Senckenberg Anatomy, Johann Wolfgang Goethe-University, Theodor Stern Kai 7, 60590 Frankfurt am Main, Germany

**Keywords:** Neuroanatomy, Cerebral arteries, Anterior cerebral artery, Recurrent artery of Heubner, Digital subtraction angiography

## Abstract

**Background:**

The anatomy of the proximal anterior cerebral artery (ACA) and its branches, including the recurrent artery of Heubner (RAH) and medial lenticulostriate arteries (MLSAs), is known for frequent variations. Impairment of these branches can result in severe consequences, including neurological deficits or cognitive impairment. This study aimed to analyze these branches and their variations in vivo, using data from 3D rotational angiographies (3D-RA).

**Material and Methods:**

We reviewed 3D-RAs of 209 hemispheres from 191 patients with pathologies remote from the region of interest. The presence, origin and course of the RAH were investigated. Delineation, origin and number of perforator vessels originating from the A1 segment (MLSAs) were assessed.

**Results:**

The RAH was observed in 151 hemispheres (72%), including a single RAH in 144 (69%) and a doubled RAH in 7 (3%) hemispheres. In 37% of cases, the RAH originated from the A1 segment, in 56% from the A1/A2 transition, and in 7% from the A2 segment. In the presence of RAH, additional MLSAs originating from the A1 segment were present in 25% of hemispheres. A weak negative correlation was identified between the presence of one or two RAH and the frequency of additional MLSAs.

**Conclusion:**

The study revealed significant differences in the presence and anatomical course of RAH and MLSAs compared to previous research. The findings highlight the importance of 3D-RA in visualizing the complex anatomy of the ACA, particularly to avoid complications during surgical or endovascular procedures.

## Introduction

The anatomy of the proximal anterior cerebral artery (ACA), i.e., the A1 segments and A2 segments and its perforators, is complex. The region supplied by the ACA and its branches is considered more variable compared to regions supplied by the middle and posterior cerebral arteries [1]. This anatomical variability is of clinical relevance, as it affects the vascular supply of important brain structures such as the basal ganglia, the internal capsule, and the frontal lobe. The proximal ACA and its branches are predisposed to a variety of vascular pathologies, such as aneurysms or vasoconstrictive disorders. Knowledge of the ACA anatomy, and its individual variations is crucial for the accurate diagnosis and treatment of vascular diseases.

Of particular importance are the recurrent artery of Heubner (RAH) and the medial lenticulostriate arteries (MLSAs), which arise from the proximal ACA and supply deep subcortical structures including the head of the caudate nucleus and anterior limb of the internal capsule (Table 1). Infarcts in these regions—often due to injury or occlusion of these small arteries—may cause significant neurologic deficits (Table 1). Therefore, accurate identification of the RAH and MLSAs is critical for preoperative risk assessment, treatment planning, and avoiding ischemic complications in both microsurgical and endovascular interventions.

**Table 1:** A1-branches: their synonyms, supplied anatomical structures and neurologic syndromes resulting from perfusion impairment. A1: A1 segment of the anterior cerebral artery (ACA), A2: A2 segment of the ACA, AcomA: anterior communicating artery

Vascular branch	Synonyms	Site of origin	Branches and supplied anatomical structures	Associated neurological symptoms
*Recurrent artery of Heubner:* described by Johann Otto Leonhard Heubner in 1872 [9] first named “Heubner’s artery” by Aitken in 1909 [10]	(long) Telencephalic artery [11] Distal medial striate artery [12] Long central artery [13] Rostral striate artery [14] Largest vessel of the medial lenticulostriate arteries [5]	A1 (3.55–6.2%) A1/A2 junction (43.4–79.2%) A2 (14.6–47.8%)[2, 15]	*Branches [*16*]:* Anterior perforating substance branches Sylvian fissure branches Olfactory branches Frontobasal branches Hypothalamic branches Optic tract branches *Anatomical structures *[5, 12, 17–19]: Internal capsule Nucleus accumbens Basal nucleus of Meynert Orbitofrontal cortex Caudate nucleus Globus pallidus Putamen Olfactory tubercle, olfactory tract Optic tract, optic chiasm Hypothalamus Diagonal band of Broca	[20]: Hemiparesis with fasciobrachiocrural predominance Aphasia in dominant hemisphere involvement Dysarthria (involvement of corticolingual and corticostriatocerebellar pathways) Choreoathetosis Visual neglect Behavioral changes such as hyperactivity or abulia (interruptions between pathways of associative cortex with deep cortical regions)
*Medial perforating arteries *[21]	Medial central arteries [21] Anteromedial central arteries (if originating from AcomA) [22] Rami diencephalica inferiores anteriores [30]	AcomA [21, 22]	*Anatomical structures [*21, 23*]:* Pituitary stalk Optic chiasm Preoptical region of hypothalamus Anterior commissure Genu of internal capsule Anterior part of Globus pallidus Striatum Fornix	[19, 24–27]: Cognitive impairment Memory deficits, Amnesia Visual deficits Extrapyramidal motoric disorders Hypophyseal dysfunction
*Lateral perforating arteries:* 8–12 thin branches [21]	*Medial lenticulostriate arteries* [28, 29] Anteromedial central arteries (if originating from A1) [22] Short central arteries [21] Inferior anterior diencephalic branches [30] Internal striate arteries [31]	A1 [21, 32, 33]	*Anatomical structures *[21, 23, 32–34]: Hypothalamus Head of caudate nucleus Anterior limb of internal capsule Lateral segment of globus pallidus Anterior and inferior portions of putamen Anterior perforating substance Medial part of anterior commissure	[34, 35]: Pure motor hemisyndrome Ataxic hemiparesis Pure sensory hemisyndrome dysarthria/clumsy hand syndrome Sensory-motor hemisyndromes Cognitive impairment

Previous anatomical studies of these vessels have relied on cadaveric dissections [2–6], selective catheter angiograms [7], or magnetic resonance angiography (MRA) [8], each of which has provided valuable insights, yet also has certain limitations in terms of resolution, coverage, or specimen variability. While cadaveric studies offer detailed insight into the vascular structures, they are generally no serial examinations and thus, do not represent the range of variation seen in the living population.

Angiographic techniques, such as computed tomography angiography (CTA), magnetic resonance angiography (MRA), or projection digital subtraction angiography (DSA) provide in vivo imaging of the vessels which is limited by factors such as spatial or contrast resolution and vessel overlay with difficulty in visualizing smaller branches.

In the clinical setting, contrast-enhanced 3D rotational angiography (3D-RA) including flat panel CT reconstructions are used in selected cases during catheter angiography, as it provides high-resolution, high-contrast 3D images of the arterial system. This enables detailed in vivo assessment of complex vascular anatomies and small vessels without the overlapping effects seen in other imaging techniques.

The aim of our study was to accurately characterize the proximal branches of the ACA, including the RAH and MLSAs , using reconstructions from routine 3D-RA. This method offers superior spatial resolution and the flexibility to choose different projections. By evaluating the distribution and variability of the perforating branches of the proximal ACA, we aimed to enhance the understanding of this anatomically complex region.

## Material and Methods

The study was a single-center, retrospective analysis that was approved by the local ethics committee. The study involved a review of our institutional neuroradiological database from 2014 to 2023 for patients who underwent digital subtraction angiography (DSA) of the anterior circulation including routine 3D-RA runs.

**Fig. 1:**
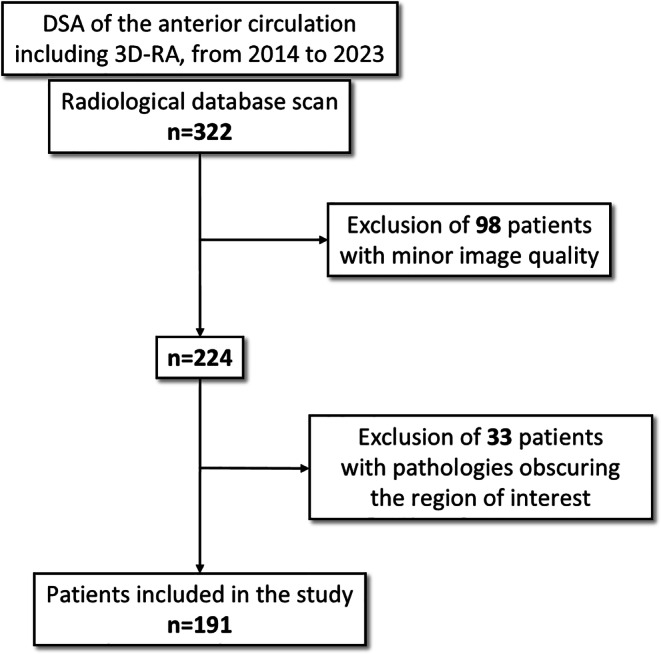
Study flowchart describing the data procurement and patient selection process for analysis

Data were obtained from 322 patients. Data from 98 patients with insufficient image quality due to motion artefacts or incomplete contrast filling were excluded. 33 patients were excluded because pathology obscured the region of interest. The remaining data from 191 patients (209 hemispheres) with adequate image quality were analyzed (Fig. 1).

DSA examinations were performed on Axiom Artis zee© biplane and Artis icono© biplane angiography systems (Siemens Healthineers, Erlangen, Germany). The angiographic systems are equipped with a flat panel detector with 2480 × 1920 pixels (2k matrix) and a pixel pitch of 154 µm × 154 µm. The spatial resolution of flat detector CT is 3.0 lp/mm with no binning and 1.5 lp/mm with 2 × 2 binning for high-resolution modes. For the 3D-RA, a native run was performed followed by a contrast-enhanced run after mechanical intraarterial injection of 20 ml of nonionic contrast material (Ultravist 240, Bayer, Berlin, Germany) into the internal carotid artery (ICA) using a flow of 3 ml/s. The injection was started 2 sec before start of the contrast-enhanced run. In each run, 133 images were obtained with a scan time of 5 sec.

3D-RA data were reconstructed using standard volume rendering and flat panel CT algorithms.

The maximum achievable spatial resolution for flat panel CT algorithms of the system used (DynaCT Micro, Siemens Healthineers, Erlangen, Germany) is up to 0.14 mm. Reconstructed datasets had spatial resolutions ranging from 0.2 to 0.3 mm isotropic. The flat panel CT-angiography reconstructions as well as 3D volume rendering reconstructions with colored window and threshold settings were used for vascular analysis.

Images were evaluated using consensus reading by two experienced neuroradiologists (12 and 33 years of practice) using a workstation with Centricity© RIS-i 7 viewer (GE Healthcare, Chicago, USA). A plugin based postprocessing was used to create custom multiplanar (MPR), maximum intensity projection (MIP) and average intensity projection views from the flat panel CT sections.

**Fig. 2:**
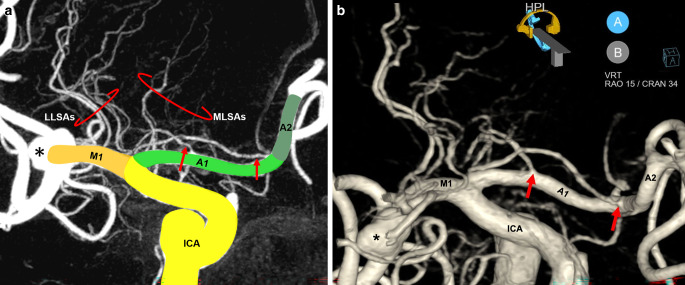
**a** Coronal angulated 3D rotational angiography (3D-RA) maximum intensity projection (MIP) (**a**) and 3D-reconstructed image (**b**) of the right anterior intracranial circulation in a patient suffering from a middle cerebral artery (MCA) bifurcation aneurysm (asterisk). Note two recurrent arteries of Heubner (RAH, arrows). A1, A2: segments of the anterior cerebral artery (ACA); M1: proximal segment of the MCA; LLSAs: lateral lenticulostriate arteries; MLSAs: medial lenticulostriate arteries

**Fig. 3:**
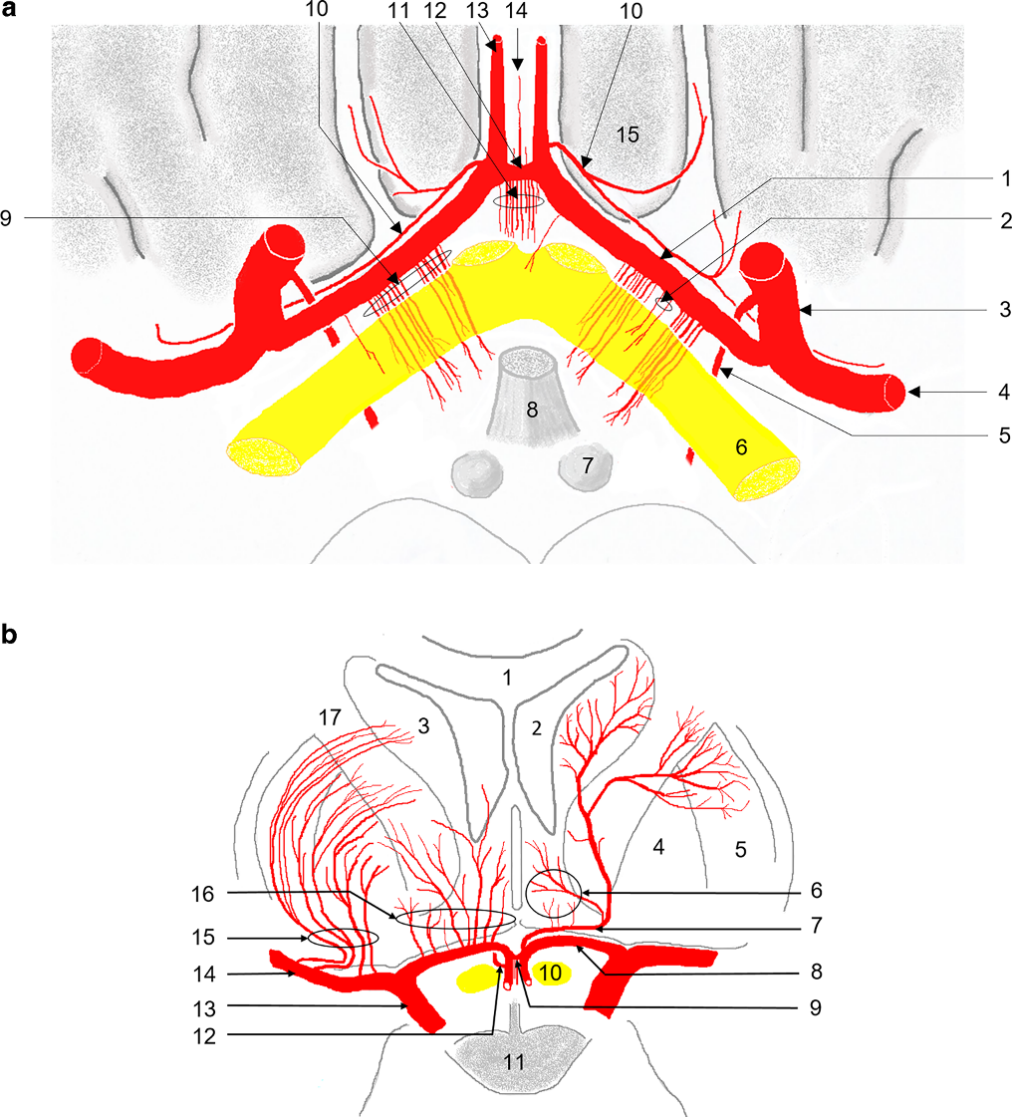
Illustration of the A1 segment and the anterior communicating artery (AcomA) showing the originating perforators and the recurrent artery of Heubner (RAH). **a** Axial view. 1: A1 segment of the anterior cerebral artery (ACA); 2: Perforators to the optical tract; 3: Internal carotid artery (ICA, C7 segment); 4: Middle cerebral artery (MCA, M1 segment); 5: Anterior choroidal artery; 6: Optical tract; 7: Mammillary bodies; 8: Hypophyseal stalk; 9: Medial lenticulostriate arteries (MLSAs); 10: Recurrent artery of Heubner (RAH) originating from the A1/A2 junction right and the proximal A2 segment left; 11: Medial perforating arteries (medial central arteries); 12: Anterior communicating artery (AcomA); 13: A2 segment of the ACA; 14: Subcallosal artery; 15: Gyrus rectus. **b** Coronal view. Semischematic illustration of the major perfusion areas of the A1 segment (left) and the recurrent artery of Heubner (RAH; right). 1: Corpus callosum; 2: Lateral ventricle; 3: Head of the caudate nucleus; 4: Globus pallidum; 5: Putamen; 6: Hypothalamic and frontobasal branches of the RAH; 7: RAH originating from the distal A1-segment; 8: A1 segment of the anterior cerebral artery (ACA); 9: Anterior communicating artery (AcomA) with subcallosal artery and medial perforating arteries; 10: Optic nerve; 11: Pituitary gland; 12: RAH originating from the proximal A2 segment; 13: Internal carotid artery (ICA); 14: Middle cerebral artery, M1 segment; 15: Lateral lenticulostriate arteries (LLSAs); 16: Medial lenticulostriate arteries (MLSAs); 17: Internal capsule

The origin of the ACA from the ICA bifurcation was identified. Delineation, origin and number of perforator vessels originating from the A1 segment of the ACA as well as the presence and course of the RAH were assessed (Figs. 2 and 3).

The vessels originating from the anterior communicating artery (AcomA) complex, i.e., the subcallosal artery and the medial perforating arteries (Fig. 3a, b), were not investigated, because adequate and complete contrast filling could not be confirmed , even after dataset fusion in patients who underwent bilateral 3D-RA.

The A1 segment was considered hypoplastic if its diameter was less 1 mm [36]. Perforating branches originating directly from the carotid‑T angle were not considered as A1 branches.

Statistical processing of the data was performed using Microsoft Excel version 16.78 (Microsoft, Redmond, WA, USA) and GraphPad Prism version 6 (GraphPad Software, San Diego, CA, USA). Spearman’s rank correlation was used for the analysis of non-parametric data.

## Results

All patients in our study underwent DSA as part of their standard diagnostic and therapeutic procedures for suspected vascular pathologies, such as arterial aneurysms and arteriovenous malformations. Patient demographics are shown in Table 2.

**Table 2:** Patient demographics.

Total number of subjects (*n*)	191
Number of hemispheres (*n*)	209
	Right: 110
	Left: 99
Age (years)	18–90 (mean: 56)
Sex	Male: 73: (38%)
	Female: 118 (62%)
Pathologies (*n*)	Verified aneurysms: 181
	Suspected aneurysm: 9
	Arteriovenous malformations: 1

### Recurrent Artery of Heubner (RAH)

A total of 158 RAHs were identified in 151 out of 209 hemispheres (72.2%). In 58 hemispheres (27.8%), no RAH was detected. In the majority of cases, specifically in 144 hemispheres (68.9%), only a single RAH was visible, while in 7 hemispheres (3.3%) two RAHs were observed. Upon analyzing the origin of the RAH, we found that 37.3% (59/158) of the RAHs originated from the A1 segment, 55.7% (88/158) from the A1/A2 junction, and 7.0% (11/158) from the proximal A2 segment.

**Fig. 4:**
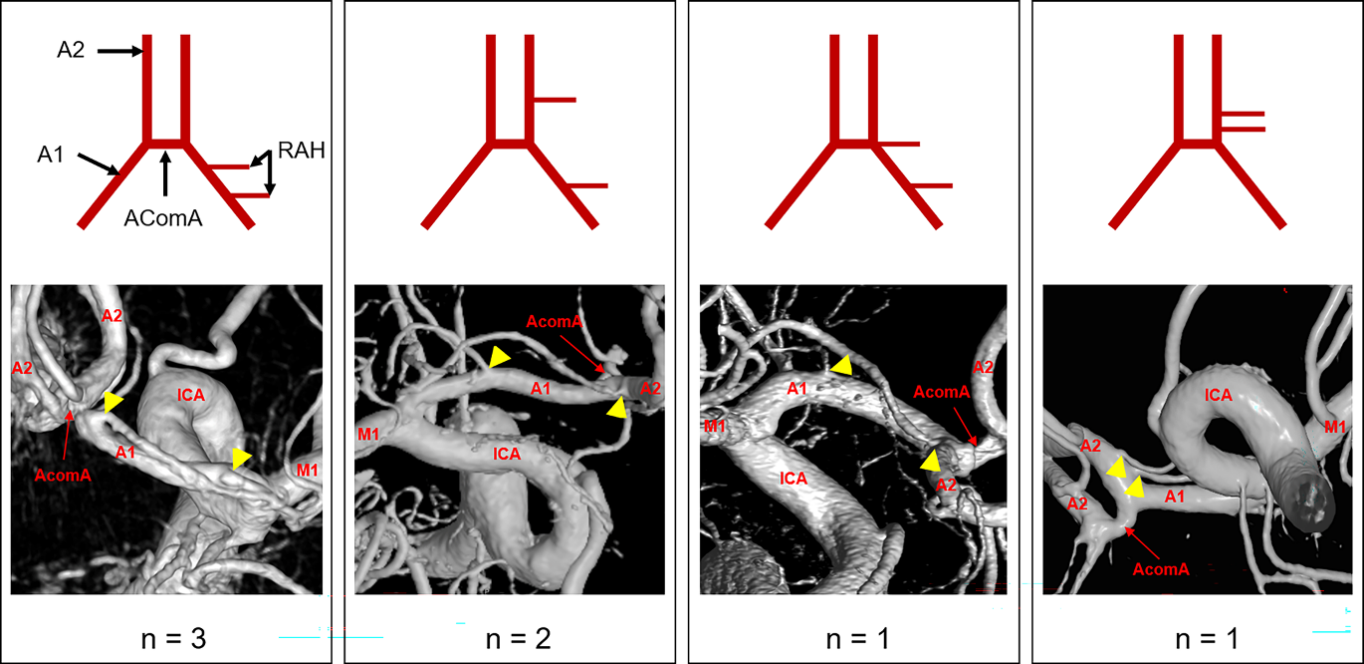
Anatomical variants of double Heubner arteries (RAH) per hemisphere and number of hemispheres in which they were observed. A1: A1 segment; A2: A2 segment, AComA: anterior communicating artery

In the seven cases with two RAHs per hemisphere, both vessels showed different origins. In three hemispheres (1.4%), both RAHs originated from the A1 segment, in two hemispheres (1.0%), one originated from the A1 segment and the other from the A1/A2 junction, in one hemisphere (0.5%), both RAHs originated from the A1/A2 junction, and in one hemisphere (0.5%), one RAH originated from the A1/A2 junction and the other from the A2 segment. A detailed representation of these cases can be seen in Fig. 4.

The direction of the RAH varied as well, with 28.5% (45/158) of RAHs directed caudally, 35.4% (56/158) cranially, 32.9% (52/158) laterally, and 3.2% (5/158) medially. The mean distance of the RAH origin from the ICA bifurcation was 11.7 mm (range: 1.0–20.0 mm), indicating considerable variability in its anatomical origin.

In a subgroup of 18 patients, 3D-DSA was performed on both hemispheres. In these cases, a single RAH was observed in one hemisphere in 6 of the 18 patients (33.3%), and no RAH was detected in the other hemisphere of another 6 out of 18 patients (33.3%). Bilateral RAHs with symmetrical origins were observed in 3 of 18 cases (16.7%), while asymmetrical bilateral RAHs were seen in 3 out of 18 other patients (16.7%).

**Fig. 5:**
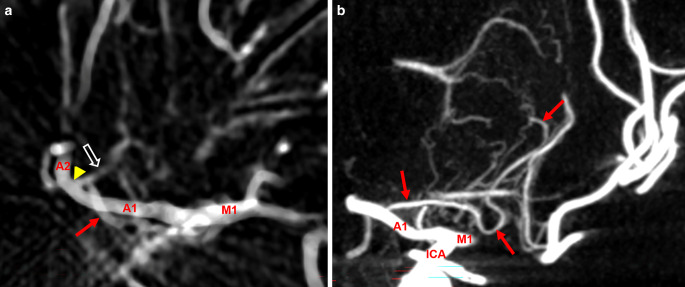
Anatomical variants of the recurrent artery of Heubner (RAH); M1: M1 segment; A1: A1 segment; ICA: internal carotid artery. **a** RAH (arrow) sharing a common origin with a frontopolar artery branch (arrowhead), average intensity projection, axial view. **b** Far lateral reaching RAH (arrows) supplying parts of the lateral basal ganglia, maximum intensity projection, coronal view

In 30 hemispheres (14.3%), the RAH was seen alongside at least one additional smaller medial lenticulostriate artery (MLSA). In one case, a common trunk for the RAH and the frontopolar artery was detected (Fig. 5a). Ten RAHs showed an elongated lateral course, partially running alongside the lateral lenticulostriate arteries (LLSAs) and extending into the basal ganglia (Fig. 5b).

**Fig. 6:**
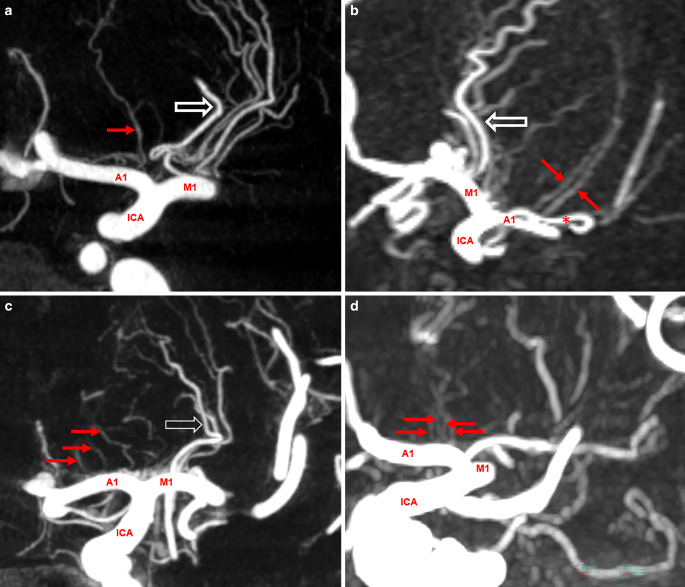
Variable numbers of medial lenticulostriate arteries (MLSAs) arising from the A1 segment. Single to four perforators (**a**–**d**, arrows) originating from the A1 segment; open arrow: LLSAs: lateral lenticulostriate arteries originating from the M1 segment

### Medial Lenticulostriate Arteries (MLSAs) Apart from RAH

Apart from the RAH, other perforating vessels originating from the A1 segment were also identified. These perforating arteries were observed in 24.9% (52/209) of hemispheres. However, multiple MLSAs were found only in 7.7% (16/209) of the hemispheres (Table 3; Fig. 6). A weak negative correlation was identified between the presence of one or two RAH and the frequency of other smaller perforating branches originating from the A1 segment (r = −0.1377; *p* = 0.0463).

**Table 3:** Number of observed medial lenticulostriate arteries (MLSAs).

Number of visible MLSAs	No. of hemispheres (%)
0	157 (75.1)
1	36 (17.2)
2	12 (5.7)
3	3 (1.5)
4	1 (0.5)

## Discussion

We analyzed a dataset consisting of 209 hemispheres from 191 patients. This study, utilizing 3D-RA, presents several novel findings compared to previous research (summarized in Table 4).

**Table 4:** Summary of anatomical studies of the recurrent artery of Heubner (RAH). DSA: digital subtraction angiography, 3D-RA: 3D rotational angiography.

Authors	Study method	No. of hemispheres	Percentage of RAHs per hemisphere [%]					Site of origin [%]			RAH origin and course
			Absent	1	2	3	4	A1	A1/A2 junction	A2	
Gaubert et al. [46]	Cadaveric	35	0	–	–	–	–	0	75	25	–
Ostrowski et al. [47]	Cadaveric	56	0	–	–	–	–	93	7		–
Westberg [48]	Cadaveric	34	3	94	3	0	0	0	100	0	–
Ahmed & Ahmed [49]	Cadaveric	24	8	79.5	12.5	0	0	0	100	0	–
Kribs & Kleihues [50]	Cadaveric	177	35	56.8	8.2	0	0	0	100	0	–
Perlmutter et al. [19]	Cadaveric	100	1	99	0	0	0	14	8	78	60% anterior
											40% superior
Dunker & Harris [51]	Cadaveric	40	0	100	0	0	0	10	55	35	–
Lemos [52]	Cadaveric	83	1.2	–	8.4	0	0	6% right 7% Ieft	73% right 67% Ieft	21% right 26% left	–
Gomes et al. [11]	Cadaveric	60	3.3	85	11.7	0	0	8	35	57	Lateral: 83%
											Superior 15%
											Lateral wall of orbitofrontal branch: 2%
Marinkovic et al. [53]	Cadaveric	66	0	73	24	3	0	17	21	34	–
Avci et al. [54]	Cadaveric	62	1.6	74.2	22.6	1.6	0	8	29	64	Superior: 38%
											Anterior: 52%
											Posterior 10%
Kang et al. [29]	3D-RA	186	46	54	0	0	0	–	–	–	–
Loukas et al. [5]	Cadaveric	69	6	77	17	0	0	14.3	62.3	23.3	–
Uzün et al. [15]	Cadaveric	108	3.7	96.3	0	0	0	6.2	79.2	14.6	–
Zunon-Kipré et al. [12]	Cadaveric	40	0	95	5	0	0	30	12	58	Superior: 31
											Anterior: 59.5
											Posterior: 4.7
Maga et al. [16]	Cadaveric	140	1.4	29.7	43.5	24.6	2.2	26.2	33.8	40	Superior: 61
											Anterior: 32
											Inferior: 4
											Posterior: 3
											Parallel to A1: 70.2
											Arching toward olfactory tract: 29.8
El Falougy et al. [2]	Cadaveric	366	5.2	88.5	6.28	0	0	3.7	45.8	50.5	–
Impiombato et al. [7]	Conventional DSA	200	88	7.7	4.3	0	0	30.4	21.7	47.8	Superior: 100
											other courses not observed “due to the incapability to determine a spatial location of a branch in a single 2D image” [7]
Matsuda et al. [55]	Cadaveric	714	1.3	96.2	2.4	0.14	0	7.4	76.3	16.3	Superior: 30.2
											Anterior: 62.1
											Posterior: 7.7
Present study	3D-RA	209	27.8	68.9	3.3	0	0	37.3	55.7	7.0	Cranial: 35.4
											Lateral: 32.9
											Caudal: 28.5
											Medial: 3.2

We found no RAH in 27.8% of hemispheres, significantly higher than the 0–5% reported in most studies, while 68.9% showed presence of a single RAH, indicating a relatively high overall occurrence.

The distribution of RAH anatomical courses reveals notable variability: 35.4% cranial, 32.9% lateral, 28.5% caudal, and 3.2% medial. These results suggest a broader range of variations than reported in earlier studies, where the RAH was most commonly described as lateral or superior. Regarding the origin of the RAH, it is significantly more likely to originate cranially (37.3%) and laterally (55.7%), with medial and caudal origins being notably less frequent. This contrasts with other studies, where superior and anterior courses predominated. Regarding the MLSAs, our study shows that in 75.1% of the hemispheres, no additional lenticulostriate arteries were visible. One additional lenticulostriate artery was seen in 17.2% of the hemispheres, two in 5.7%, three in 1.5%, and four in 0.5%.

These data highlight an interesting variance in the frequency of additional lenticulostriate arteries, which may not have been adequately accounted for in previous studies. However, discrepancies in the reported frequencies and anatomical variations—particularly when comparing our findings to radiological studies—may partly be explained by differences in spatial resolution across imaging modalities and reconstruction algorithms, which often vary by vendor. This technical variability may limit the direct comparability with previous imaging-based studies.

Usually, 0–17 thin MLSAs emerge from the A1 segment [21, 28]. In the present study, we identified up to 4 A1 perforators in approximately 25% of assessed hemispheres, coursing cranially to the region of the anterior perforating substance. The number of observable A1 perforators showed a weak inverse correlation to the presence of a RAH, which is also considered by some authors to be the largest representative of the MLSAs [2, 11, 29]. In general, the diameter of the MLSAs was reported to range between 0.05 to 1.9 mm [28]. The maximum spatial resolution of the datasets used in our study was 0.2 mm isotropic. The failure to identify MLSAs in 75% of hemispheres may thus be partly attributable to the limited spatial resolution of the datasets. The ability to visualize small intracranial vessels such as the MLSAs is therefore closely linked to the technical performance of the imaging system. The short scan time of 5 sec could also limit contrast filling of smaller branches with slow flow.

However, the limited number of MLSAs observed in this study may also be attributed to inherent anatomical differences. Kang et al. [29] described a balance between the number of MLSAs and LLSAs originating from the M1 segment (Fig. 3b). Their findings suggest that a high number of LLSAs is associated with a lower number of narrow MLSAs. This interdependency may also have contributed to the low number of MLSAs observed in our study. However, LLSAs were not specifically examined in our study, as the focus was on the ACA, and in many cases, LLSAs were not included in the field of view. As a result, a systematic examination of this balance was not feasible in our work.

Moreover, previous studies have shown that the RAH replaces additional MLSAs in approximately 26% of cases [37]. Given the high frequency of RAHs observed in our datasets, this may also explain the relatively low number of MLSAs in this study. In line with this, a weak negative correlation was observed between the presence of one or two RAHs and the frequency of other MLSAs originating from the A1 segment.

The RAH follows a recurrent course along the A1 segment and supplies key regions including parts of the basal ganglia and internal capsule (Table 1; Fig. 3). We observed at least one RAH in approximately 72% of the assessed hemispheres. Our results are consistent with the majority of cadaver studies, indicating that the RAH is commonly a single artery (Table 4). In approximately 69% of all hemispheres, a single RAH was observed. Additionally, in our study, the RAH mainly originated from the A1/A2 junction in about 55.7% of hemispheres, in line with previous studies (Table 4). The course and origin of the RAH along the A1 segment were readily identified in all cases, which is consistent with the observations of Impiombato et al., who studied standard selective angiograms [7].

We also found that the RAH originated from the A1 segment in about 36% of the hemispheres, while it originated from the A2 segment in about 8% of the hemispheres. These observations contrast with several cadaveric studies [2, 5, 15, 38], but are in accordance with the 3D-RA results of Kang et al. [29]. In his investigation MLSAs including the RAH frequently originated from the A1 segment (in 59 of 140 hemispheres; 42%). Although the RAH usually originates as a single artery, we found a double RAH with varying origins in 3.3%, supporting the notion that the number of RAHs may vary within a single hemisphere. In addition, we identified a common trunk of the RAH and the frontopolar artery from A2 in 2 cases, which is a known variant ([11, 39]; Fig. 5a).

From a clinical point of view, our results indicate that 3D-RA can be effectively used to visualize the complex anatomy of the proximal ACA prior to endovascular or surgical procedures. It is estimated that a lack of knowledge about variant anatomy causes about 10% of medical errors [40]. It is therefore critical to have an accurate understanding and visualization of individual and variant anatomy.

The RAH and other MLSAs supply important grey and white matter areas (summarized in Table 1). Impaired perfusion to these structures is commonly associated with severe neurological deficits, such as hemiparesis, aphasia, dysarthria, sensory deficits and behavioral changes, including dementia [20, 34, 35]. This highlights the importance of preserving perforating vessels, especially the RAH, during surgical or endovascular procedures, so that correct identification of these vessels during angiography helps to avoid unwanted complications. For instance, when clipping AcomA aneurysms, it may be necessary to sacrifice small frontopolar and orbitofrontal arteries, but it is crucial to identify and preserve the RAH [41].

Our study has some limitations. With current angiographic techniques and reconstruction algorithms, many but by no means all perforating arteries originating from the A1 segment can be visualized, as spatial resolution is limited. However, the objective of our study was to investigate whether routine 3D-RAs are sufficient to demonstrate variants of the RAH and major perforating branches. Previous studies have found that 3D-reconstructed images tend to overlap closely spaced vascular structures, which can make it difficult to accurately identify the vessels of interest. To address this issue, we employed flat-panel CT reconstructions—currently the best available option for high-resolution imaging of small vessels—which, as shown in previous studies, have also proven useful in resolving ambiguous cases [42, 43].

Further improvement of spatial resolution in imaging techniques could help overcome current limitations. For example, improvements in high-resolution post-contrast MRI angiography at 7 T [8] are likely to gain importance in this field of research, alongside conventional angiography.

It is also important to consider that the frequency of the RAH in our study may be overestimated. In patients with presumed arterial pathologies at the AcomA, the side with the larger caliber A1 is usually injected for 3D-RA to obtain optimal contrast enhancement of the AcomA. As a result, narrow-caliber hypoplastic A1 segments with small-caliber or absent RAHs may be underrepresented in our dataset. Furthermore, we deliberately excluded the AcomA and its branches, such as the subcallosal artery and medial perforating arteries, as contrast enhancement may be insufficient depending on the diameter and direction of flow. Several previous studies have investigated the AComA complex and its perforating branches in greater detail from clinical and imaging standpoints, including approaches using angiography or 3D-RA [42, 44, 45]. These studies provide important insights into the anatomy and variability of this region, and future investigations using novel high-resolution flat-panel CT systems or time-resolved 3D-RA may further enhance the understanding of the subcallosal artery and other AcomA branches.

As summarized in Table 4, most research on the neuroanatomy of the ACA perforators are based on studies of cadaveric brains. However, these methods are also associated with certain limitations. If the dye is injected at too high a pressure, small vessels integrity may be compromised, whereas insufficient pressure prevents complete dye filling of small vascular structures. Narrow caliber vessels, in particular, may be missed due to this methodological limitation. Moreover, the cadaver studies vary in the number of RAHs they report (Table 4). Differences in the counting rules used by the authors may account for some of the heterogeneity of these studies.

In conclusion, the 3D-RA method provides novel insights into the high variability of A1 segment branches, particularly the RAH, with greater precision and a broader range of variations compared to older, predominantly cadaveric studies. These findings underline the feasibility of reconstructions from routine 3D-RA in depicting the neurovascular anatomy and its variants, which may be particularly relevant for decision-making prior to treating pathologies in this region.
